# ^68^Ga-FAPI-04 PET/MR is helpful in differential diagnosis of pancreatitis from pancreatic malignancy compared to ^18^F-FDG PET/CT: a case report

**DOI:** 10.1186/s41824-021-00106-1

**Published:** 2021-06-15

**Authors:** Yi Shou, Qiaoyi Xue, Jianmin Yuan, Jun Zhao

**Affiliations:** 1 Department of Nuclear Medicine, Shanghai East Hospital, School of Medicine, Tongji University, Shanghai, China; 2 Central Research Institute, UIH Group, Shanghai, China

**Keywords:** ^68^Ga-FAPI, PET/MR, PET/CT, Pancreatitis, Pancreatic cancer, IgG4-RD

## Abstract

**Introduction:**

^68^Ga-fibroblast activation protein-specific enzyme inhibitor 04 (FAPI-04) is a radiolabelled molecular agent targeting the inhibitor of fibroblast activation protein (FAP), which is often present in tumor stroma and inflammatory tissue with prominent fibroblast proliferation. FAPI-04 is a promising PET tracer for tumor imaging as well as IgG4-related disease (IgG4-RD).

**Case description:**

We herein present a case where ^68^Ga-FAPI PET/MR helped to diagnose IgG4-RD that involved pancreas and bile duct. A 62-year-old patient suffered from diffusive discomfort at middle upper abdomen and presented brown urine. Blood test revealed abnormal liver function and elevated IgG4 (4.830g/L↑). ^18^F-FDG PET showed enlarged uncinate process and dilated bile duct tree. Mild increase of FDG uptake in uncinate process and head of pancreas indicated possible pancreatic malignancy, but the clinical evidence was not sufficient and histology examination was negative. ^68^Ga-FAPI PET revealed prominent increased radioactivity distribution in the entire pancreas and bile duct, suggesting IgG4-RD.

**Conclusion:**

FAPI-04 is not only a good PET imaging tracer for tumors, but also for prominent fibroblast-mediated inflammation. FAPI imaging should be considered when the diagnosis using ^18^F-FDG imaging is ambiguous. The presented case illustrates that ^68^Ga-FAPI-04 PET is helpful in improving the differential diagnosis of pancreatitis and pancreatic cancer.

## Introduction

^18^F-FDG PET/CT and PET/MR imaging are gaining increasing use in clinical settings, but their applications are limited in the differential diagnosis of inflammatory or malignant pancreatic diseases (Nguyen et al., 2011). This is mainly due to the fact that ^18^F-FDG is a non-specific imaging agent. In most cases, both tumorous and inflammatory disease show high radioactivity, while in other cases, tumor lesions and inflammatory lesions show mild or normal radioactivity in the involved organs. The limitation of ^18^F-FDG renders the differential diagnosis challenging for neoplastic diseases and inflammatory diseases. For instance, when autoimmune pancreatitis appears to be hypermetabolic in a focal area, it might be misinterpreted as pancreatic malignancy (Nguyen et al., 2011; Zheng et al., 2018).

^68^Ga-FAPI-04 is a newly developed tumor imaging tracer which targets fibroblast activation protein (FAP) (Loktev et al., 2018; Lindner et al., 2018). Unlike ^18^F-FDG that accumulates in both cancer cells and active inflammatory lesions due to upregulation of glycolytic flux, uptake of FAPI is directly associated with the degree of fibrosis (Luo et al., 2020a). Therefore, when ^18^F-FDG imaging reveals inconclusive information for differentiating inflammation from tumor lesions, FAPI imaging may provide important clue and yield more accurate diagnosis.

## Case description

**Fig. 1:**
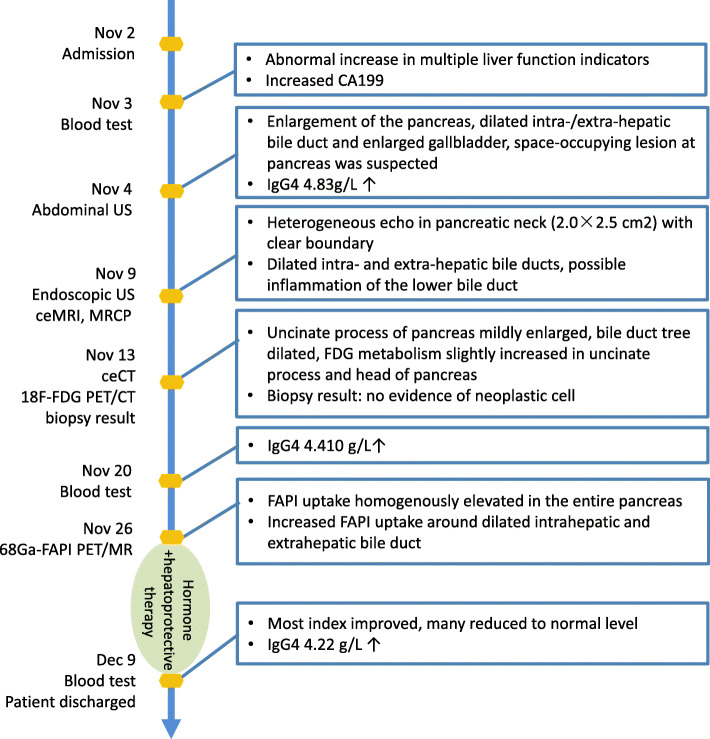
Timeline of events

A 62-year-old Asian man who had diffusive discomfort in the middle upper abdomen, accompanied by brown urine, mild yellow sclera, and occasional malaise was admitted to our hospital on November 2, 2020. Timeline of events is shown in Fig. 1.

Routine blood test (November 3, 2020) showed abnormal liver function: total bilirubin 43.00 umol/L↑(≤ 26.0 umol/L), direct bilirubin 41.0 umol/L↑(≤ 8.0 umol/L), total bile acid 61.0 umol/L↑(0-10.0 umol/L), glycocholic acid 64.5 mg/L↑(0-10.0mg/L), alanine aminotransferase 559U/L↑ (9-50 U/L), aspartate aminotransferase 401U/L↑ (15-40 U/L), AST mitochondrial isozyme 32.3 U/L↑ (≤15 U/L), alkaline phosphatase 808 U/L↑(45-125 U/L), γ-glutamyl transpeptidase 1799 U/L↑(10-60 U/L), lactate dehydrogenase 327 U/L↑ (120-250 U/L), and superoxide dismutase 259 U/mL↑ (129-216 U/mL). Serum tumor marker test showed elevated CA199 43.9 U/ml↑(≤30 U/ml). Hepatitis B and hepatitis C tests were negative. Serum IgG4 testing was performed to screen for autoimmune hepatitis, and the result was 4.830g/L↑ (< 2.01g/L).

Routine abdominal ultrasound (November 4, 2020) showed diffusive slight enlargement of the pancreas, dilated intra-/extra-hepatic bile duct, and enlarged gallbladder; space-occupying lesion at pancreas was detected.

**Fig. 2:**
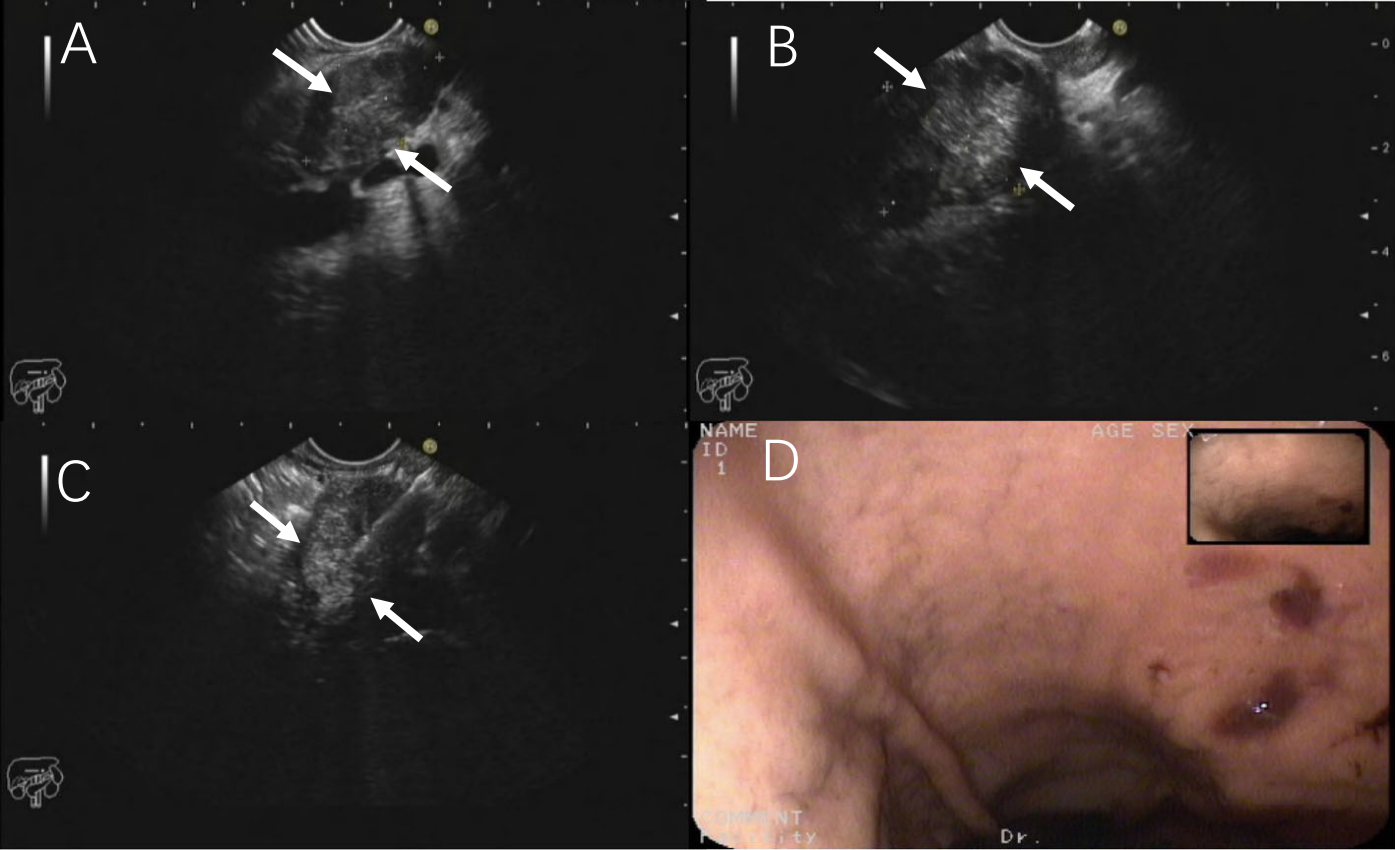
Endoscopic ultrasound showed diffuse enlargement of the pancreas, dilated intra-/extra-hepatic bile duct, and enlarged gallbladder; occupancy of the head of the pancreas was suspected

**Fig. 3:**
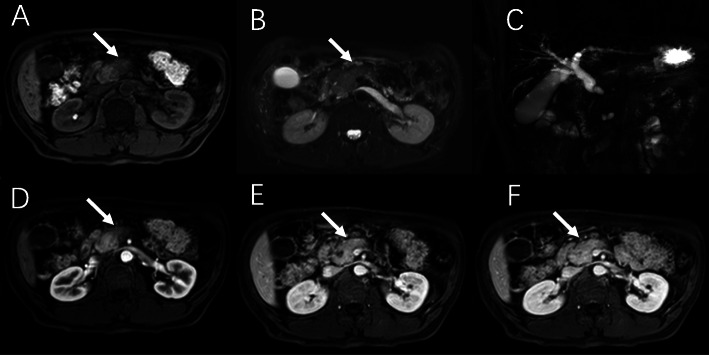
Contrast enhanced MR and MRCP showed that the intrahepatic bile duct was slightly dilated, common bile duct was dilated, and inflammation of lower segment of common bile duct was possible

Endoscopic ultrasound (EUS) and MRI were performed on November 9, 2020. EUS showed uneven echo in pancreatic neck adjacent to the portal vein and superior mesenteric vein (size about 2.0×2.5 cm^2^) with clear boundary. No obvious expansion of the main pancreatic duct in the body and tail pancreas was found. Possible malignancy was considered (Fig. 2). EUS-guided fine needle aspiration (FNA) was performed on the lesions at the neck of the pancreas via a transgastric approach. A small amount of white substance and some dark red suspension were collected. Cytological smear, liquid-based cytology, and histopathological examinations were performed respectively. Enhanced abdominal MRI and magnetic resonance cholangiopancreatography (MRCP) showed that the intrahepatic bile duct was slightly dilated, common bile duct was dilated, inflammation of lower segment of common bile duct was suspected, and the malignancy could still not be ruled out (Fig. 3).

**Fig. 4:**
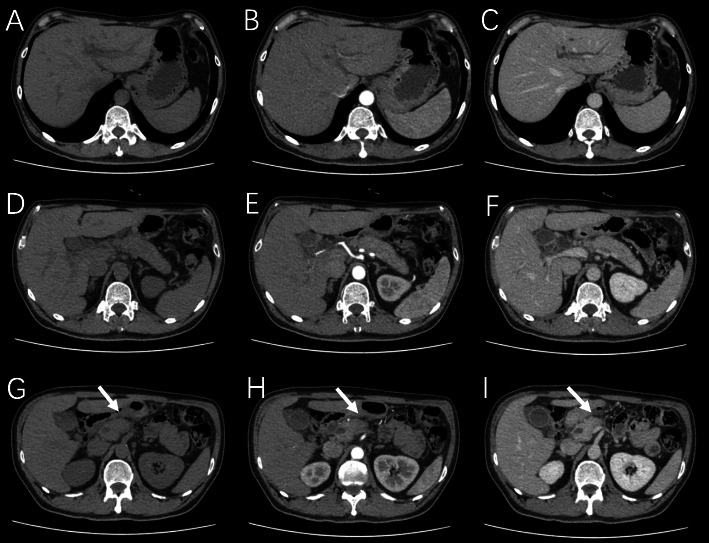
Thin-slice enhanced CT scan of pancreas showed the uncinate process of pancreatic head lesion, suggesting possible malignancy with intrahepatic and extrahepatic bile duct dilatation

**Fig. 5:**
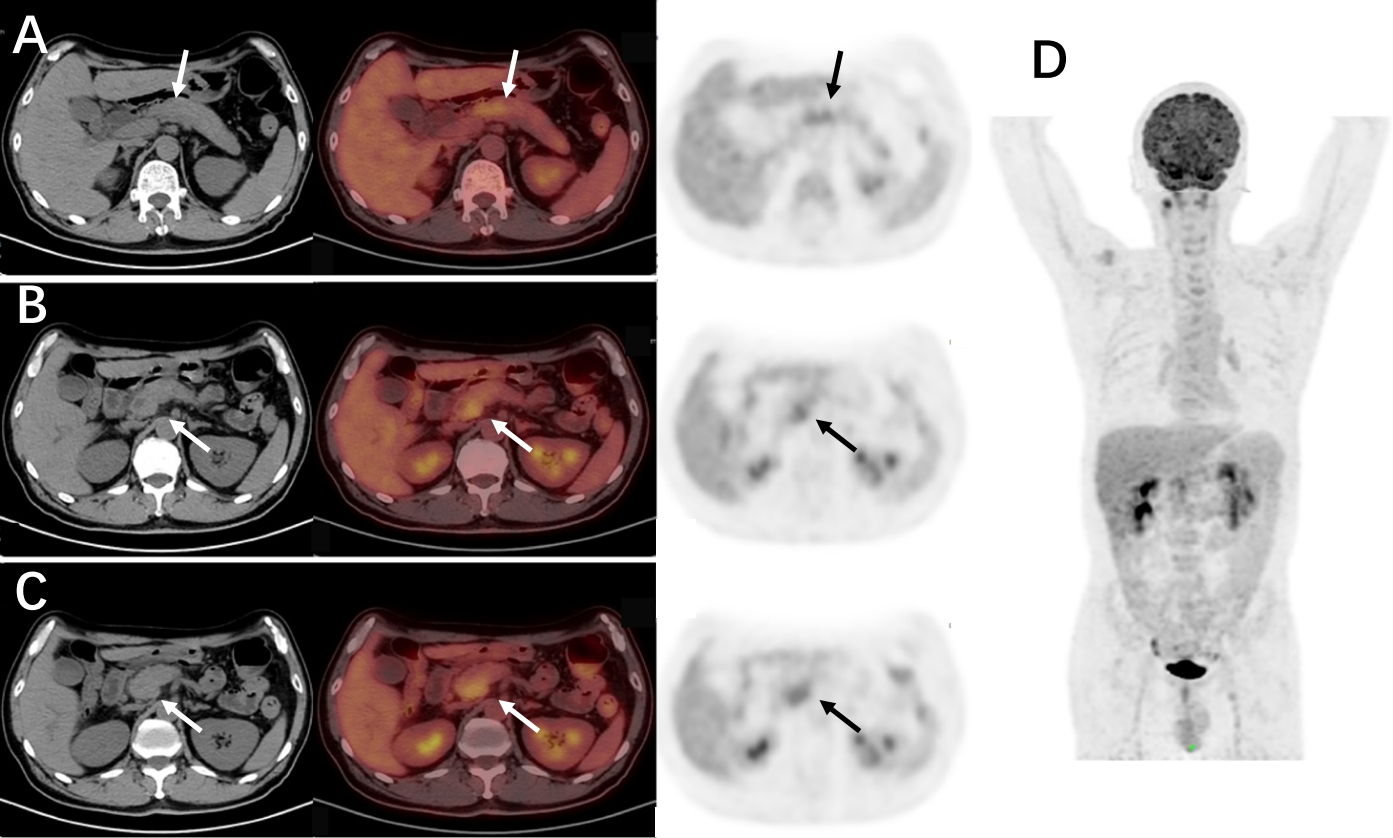
^18^F-FDG PET/CT images of IgG4-RD; the mild increase of FDG uptake in uncinate process and neck of pancreas was difficult to differentiate from pancreatic cancer

In order to identify the nature of pancreatic lesions, further thin-slice enhanced CT and ^18^F-FDG PET/CT were performed on November 13, 2020. CT showed the suspected lesion in uncinate process of pancreas with intrahepatic and extrahepatic bile duct dilatation, suggesting possible malignancy (Fig. 4). ^18^F-FDG PET/CT showed the uncinate process of pancreas was mildly enlarged with bile duct tree dilated, the FDG metabolism was slightly increased in uncinate process and head of pancreas (SUV_max_=4.07, SUV_mean_=2.25, 2.4cm × 1.8cm), and the possibility of malignant tumor was considered (Fig. 5).

On November 13, the result of FNA of pancreatic neck lesion turned out to be malignant negative, no evidence of neoplastic cell was seen in cytology and histopathological examination, and re-examination of serum IgG4 was 4.410 g/L↑ (< 2.01 g/L).

**Fig. 6:**
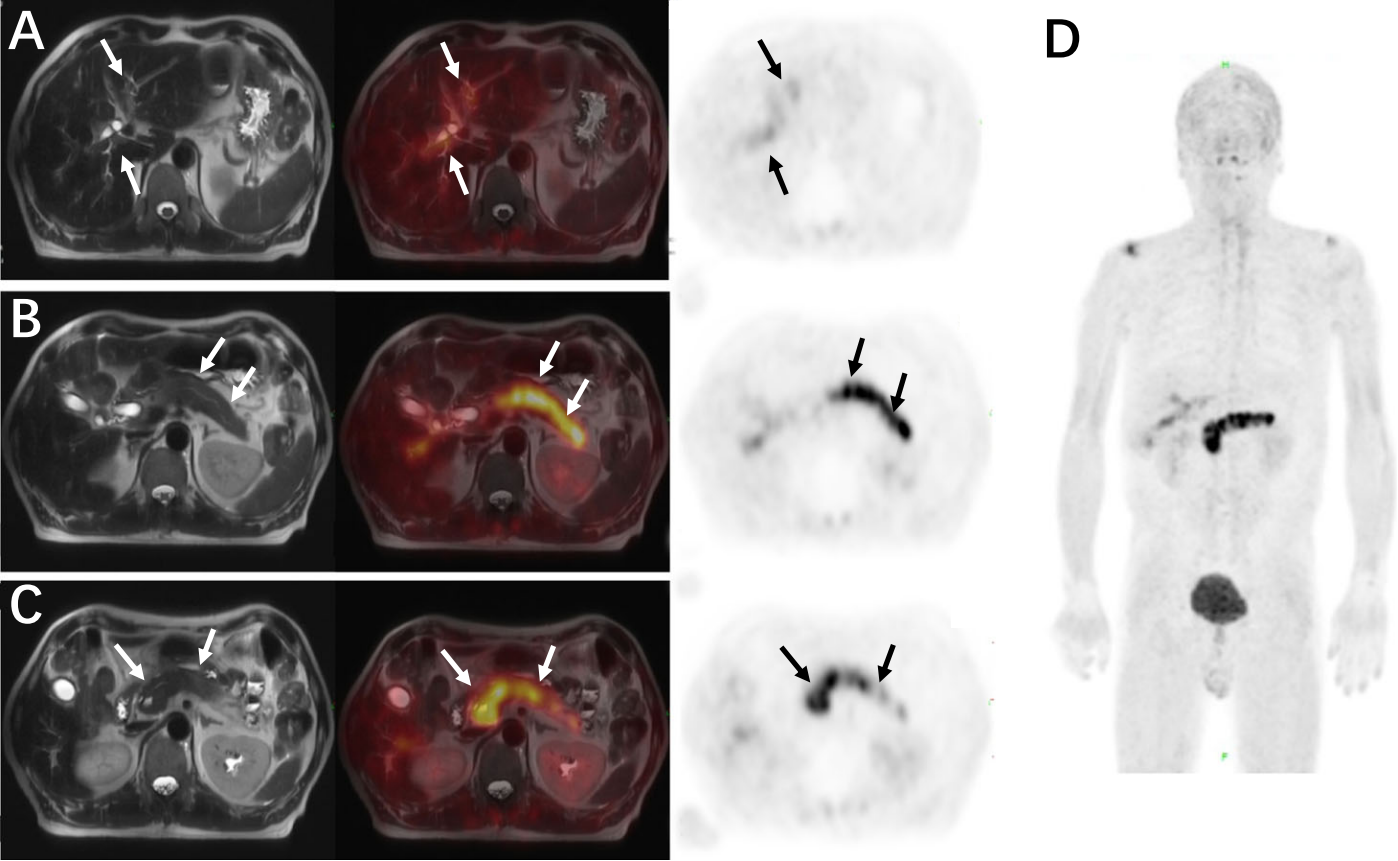
^68^Ga-FAPI PET/MR images of IgG4-RD, the intensely increased ^68^Ga-FAPI uptake in the entire pancreas, and dilated bile duct made unequivocally diagnosis of IgG4-RD

Up to this point, common pathogenesis for abnormal liver function (such as alcoholic, viral, pharmacological, genetic factors) was excluded. Based on elevated IgG4 and negative biopsy histology, IgG4-related disease was considered. Meanwhile, various imaging modalities were highly suggestive of underlying malignant tumor lesions. For further differentiation, the patient was enrolled in the clinical trial of ^68^Ga-FAPI PET/MR imaging approved by the institutional review board in our hospital, and written informed consent was obtained from the patient. From ^68^Ga-FAPI PET/MR, homogenously elevated radioactivity uptake was found in the entire pancreas (SUV_max_=11.04, SUV_mean_=6.15) and increased radioactivity was also found around dilated intrahepatic and extrahepatic bile duct (SUV_max_=3.61, SUV_mean_=1.92) (Fig. 6). These findings provided evidence for diagnosis of IgG4-RD involving pancreas and biliary tract.

Hormone therapy was initiated thereafter. Specifically, the patient was treated with methylprednisolone and prednisolone, as well as medication for liver protection, bile evacuation, and nutritional support.

After 2 weeks’ treatment, the patient’s blood test was as follows: total bilirubin 17.20 umol/L (≤26.0 umol/L), direct bilirubin 14.3 umol/L↑ (≤8.0 umol/L), total bile acids 11.7 umol/L↑(0–10.0 umol/L), glycocholic acid 6.6 mg/L (0–10.0 mg/L), alanine aminotransferase 41 U/L (9–50 U/L), aspartate aminotransferase 18 U/L (15–40 U/L), AST mitochondrial isoenzyme 1.7 U/L (≤15 U/L), alkaline phosphatase 338 U/L↑ ( 45–125 U/L), γ-glutamyl transpeptidase 478 U/L↑ (10–60 U/L), lactate dehydrogenase 116 U/l (120–250 U/l), and superoxide dismutase 192 U/mL (129–216 U/mL). Tumor marker CA199 was 10.4 U/ml (≤30 U/ml) and IgG4 was 4.220 g/L↑ (<2.01 g/L). The patient’s clinical symptoms improved significantly and liver function improved, so he was discharged on December 9, 2020.

## Discussion

^18^F-FDG PET has demonstrated advantages over other anatomical imaging modalities (such as CT, MR, B-ultrasound), but there are still challenges in differentiating autoimmune pancreatitis from pancreatic cancer (Luo et al., 2020a), especially in focal autoimmune pancreatitis when there is no indication of inflammation involved in other related organs (such as salivary glands, orbit, thyroid, lung, retroperitoneal, kidney, lymph node) (Zhang et al., 2014). ^68^Ga-FAPI is a radiolabelled agent targeting FAP, which is often present in tumor stroma (Loktev et al., 2018; Lindner et al., 2018), in addition to inflammatory tissue with prominent fibroblast proliferation as plasma cell-mediated sclerosing inflammation (Luo et al., 2020a).

Our finding of FAPI PET imaging is in line with previously reported cases. Luo et al. published a case of IgG4-RD revealed by ^18^F-FDG and ^68^Ga-FAPI, where both FDG and FAPI showed intense radioactivity at parotid gland, submandibular gland, and the pulmonary lesions, but FAPI showed intense uptake in the uncinate process of the pancreas that was not shown in FDG PET. The authors concluded that although ^68^Ga-FAPI was not more tumor-specific than FDG, it might be more sensitive than FDG in detecting IgG4-RD (Luo et al., 2019). Another case reported by Pan et al. compared FDG with FAPI in a recurrent IgG4-RD. They found that most of the lesions shown in ^18^F-FDG PET/CT were also FAPI-avid, but ^68^Ga-FAPI also accumulated in the lacrimal glands that were missed by FDG PET (Pan et al., 2020). These positive findings of ^68^Ga-FAPI highlighted that ^68^Ga-FAPI may provide important complementary information in the evaluation of IgG4-RD.

In our case, ^68^Ga-FAPI PET showed the inflammation involving the entire pancreas and bile duct tree, which was not detected by ^18^F-FDG PET. This case confirmed the promising value of ^68^Ga-FAPI in the evaluation of IgG4-RD. FDG, as an inflammatory imaging agent, can effectively reveal tissues with inflammatory cell infiltration; however, for tissues with prominent fibroblast mediated inflammation, FAPI may be more sensitive. If malignancy suspected lesion is FDG-avid, FAPI-avid is highly suggestive of malignancy, whereas FAPI-non-avid may imply acute inflammation that does not trigger fibroblast proliferation. Diagnosis becomes challenging when suspected malignancy is found FDG-non-avid. That is where FAPI imaging is recommended, as non-avid FAPI uptake may suggest chronic fibrosis.

A pitfall worth mentioning is that one type of pancreatic cancer, the pancreatic ductal adenocarcinoma (PDAC), expresses prominent FAP in the cancerous tissue and thus may have similar image presentation with IgG4-RD when using ^68^Ga-FAPI (Röhrich et al., 2020). For instance, Luo et al. published a case where FAPI uptake in inflammation masked tumor activity in pancreatic cancer (Luo et al., 2020b). Tumor-induced pancreatitis and cholangitis that show increased radioactivity may complicate FAPI uptake of the tumor itself. Therefore, it is crucial to comprehensively evaluate specific organs involved in hyper-radioactivity, the homogeneity of FAPI uptake, the contour and density/signal of the lesion in CT /MR images, and the clinical manifestations.

## Conclusion

In this case, inflammation in the pancreas mimicked focal malignant lesions in ^18^F-FDG imaging, but ^68^Ga-FAPI-04 imaging showed uniform increased uptake throughout the pancreas, which ruled out potential malignancy and confirmed the IgG4-RD diagnosis. This case demonstrated that ^68^Ga-FAPI-04 PET is more sensitive to IgG4-RD compared with ^18^F-FDG, and thus may be helpful in improving the differential diagnosis of pancreatitis and pancreatic cancer.

## Data Availability

The datasets used or analyzed during the current study are available from the corresponding author on reasonable request.

## Abbreviations

IgG4-RD: Immunoglobulin G4-related disease
MRCP: Magnetic resonance cholangiopancreatography
PET/CT: Positron emission tomography/computed tomography
PET/MR: Positron emission tomography/magnetic resonance
FDG: Fluorodeoxyglucose
FAP: Fibroblast activation protein
FAPI: FAP-specific enzyme inhibitor
EUS: Endoscopic ultrasound
US-FNA: Ultrasound-guided fine needle aspiration

## References

[CR1] Lindner T, Loktev A, Altmann A, Giesel F, Kratochwil C, Debus J, Jäger D, Mier W, Haberkorn U (2018). Development of quinoline-based theranostic ligands for the targeting of fibroblast activation protein. J Nucl Med.

[CR2] Loktev A, Lindner T, Mier W (2018). A tumor-imaging method targeting cancer-associated fibroblasts. J Nucl Med.

[CR3] Luo Y, Pan Q, Zhang W (2019). IgG4-related disease revealed by 68Ga-FAPI and 18F-FDG PET/CT. Eur J Nucl Med Mol Imaging.

[CR4] Luo Y, Pan Q, Yang H, Peng L, Zhang W, Li F (2020). Fibroblast activation protein targeted PET/CT with 68Ga-FAPI for imaging IgG4-related disease: comparison to 18F-FDG PET/CT. J Nucl Med.

[CR5] Luo Y, Pan Q, Zhang W, Li F (2020). Intense FAPI uptake in inflammation may mask the tumor activity of pancreatic cancer in 68Ga-FAPI PET/CT. Clin Nucl Med.

[CR6] Nguyen VX, Nguyen CC, Nguyen BD (2011). 18FDG PET/CT imaging of the pancreas: spectrum of diseases. Journal of the Pancreas.

[CR7] Pan Q, Luo Y, Zhang W (2020). Recurrent immunoglobulin G4-related disease shown on 18F-FDG and 68Ga-FAPI PET/CT. Clin Nucl Med.

[CR8] Röhrich M, Naumann P, Giesel FL et al (2020) Impact of 68Ga-FAPI-PET/CT imaging on the therapeutic management of primary and recurrent pancreatic ductal adenocarcinomas. J Nucl Med:jnumed.120.253062. 10.2967/jnumed.120.253062 10.2967/jnumed.120.253062 PMC8729866 33097632

[CR9] Zhang J, Chen H, Ma Y, Xiao Y, Niu N, Lin W, Wang X, Liang Z, Zhang F, Li F, Zhang W, Zhu Z (2014). Characterizing IgG4-related disease with 18F-FDG PET/CT: a prospective cohort study. Eur J Nucl Med Mol Imaging.

[CR10] Zheng L, Xing H, Li F, Huo L (2018). Focal autoimmune pancreatitis mimicking pancreatic cancer on FDG PET/CT imaging. Clin Nucl Med.

